# Introduction of a hydrolysis probe PCR assay for high-throughput screening of methicillin-resistant *Staphylococcus aureus* with the ability to include or exclude detection of *Staphylococcus argenteus*

**DOI:** 10.1371/journal.pone.0192782

**Published:** 2018-02-09

**Authors:** Katja Bogestam, Martin Vondracek, Mattias Karlsson, Hong Fang, Christian G. Giske

**Affiliations:** 1 Department of clinical microbiology, Karolinska University Hospital, Stockholm, Sweden; 2 Division of microbiology, Department of Laboratory Medicine, Karolinska Institutet, Stockholm, Sweden; Universitatsklinikum Munster, GERMANY

## Abstract

Many countries using sensitive screening methods for detection of carriage of methicillin-resistant *Staphylococcus aureus* (MRSA) have a sustained low incidence of MRSA infections. For diagnostic laboratories with high sample volumes, MRSA screening requires stability, low maintenance and high performance at a low cost. Herein we designed oligonucleotides for a new *nuc* targeted hydrolysis probe PCR to replace the standard in-house *nuc* SybrGreen PCR assay. This new, more time-efficient, PCR assay resulted in a 40% increase in daily sample capacity, with maintained high specificity and sensitivity. The assay was also able to detect *Staphylococcus aureus* clonal cluster 75 (CC75) lineage strains, recently re-classified as *Staphylococcus argenteus*, with a sensitivity considerably increased compared to our previous assay. While awaiting consensus if the CC75 lineage of *S*. *aureus* should be considered as *S*. *argenteus*, and whether methicillin-resistant *S*. *argenteus* should be included in the MRSA definition, many diagnostic laboratories need to update their MRSA assay sensitivity/specificity towards this lineage/species. The MRSA screening assay presented in this manuscript is comprised of *nuc* oligonucleotides separately targeting *S*. *aureus* and CC75 lineage strains/*S*. *argenteus*, thus providing high user flexibility for the detection of CC75 lineage strains/*S*. *argenteus*.

## Introduction

Methicillin resistant *Staphylococcus aureus* (MRSA) is a multidrug resistant pathogen that causes both nosocomial and community-acquired infections worldwide. Rapid and accurate detection of MRSA is essential to enable early treatment and prevent further transmission of this pathogen. In Sweden, a relatively high percentage of patients admitted to hospitals are screened for the presence of MRSA. Although it is not possible to show a causative link between rigorous screening and the low incidence of MRSA in Sweden, it is still believed that the screening strategy has contributed to the favorable situation [1]. As the largest diagnostic laboratory in northern Europe, this strategy results in high sample volumes with high demands in efficiency at low costs.

MRSA is often diagnosed by selective plating and/or by molecular assays, targeting *S*. *aureus* specific genes and genes carrying antibiotic resistance. To further increase sensitivity, a pre-enrichment step can be included [2, 3]. By using molecular based assays, including polymerase chain reaction (PCR), large sample volumes can be screened for MRSA within a few hours.

A common MRSA screening PCR, used both for in-house and commercial assays, is the detection of the staphylococcal cassette chromosome *mec* (SCC*mec*) that in one PCR reaction can detect both the *S*. *aureus* specific *orfX* area and the mobile cassette carrying antibiotic resistance genes. However, while the *S*. *aureus* core genome is stable, the mobile genetic elements, on which the antibiotic resistance genes lie, are more variable [4]. Failure to detect certain MRSA strains in screening can result in episodes of strain transmission in healthcare facilities. Several studies have in fact shown that the SCC*mec* PCR assay can generate false negative results due to SCC*mec* variants [5, 6] as well as false positive results in *S*. *aureus* lacking the antibiotic resistance gene *mecA* [7, 8]. Commercial PCR systems utilizing the detection of SCC*mec* are available yet expensive, while in-house assays detecting SCC*mec* require frequent oligonucleotide updates by the user due to the polymorphism within the cassette [9].

**Fig 1:**
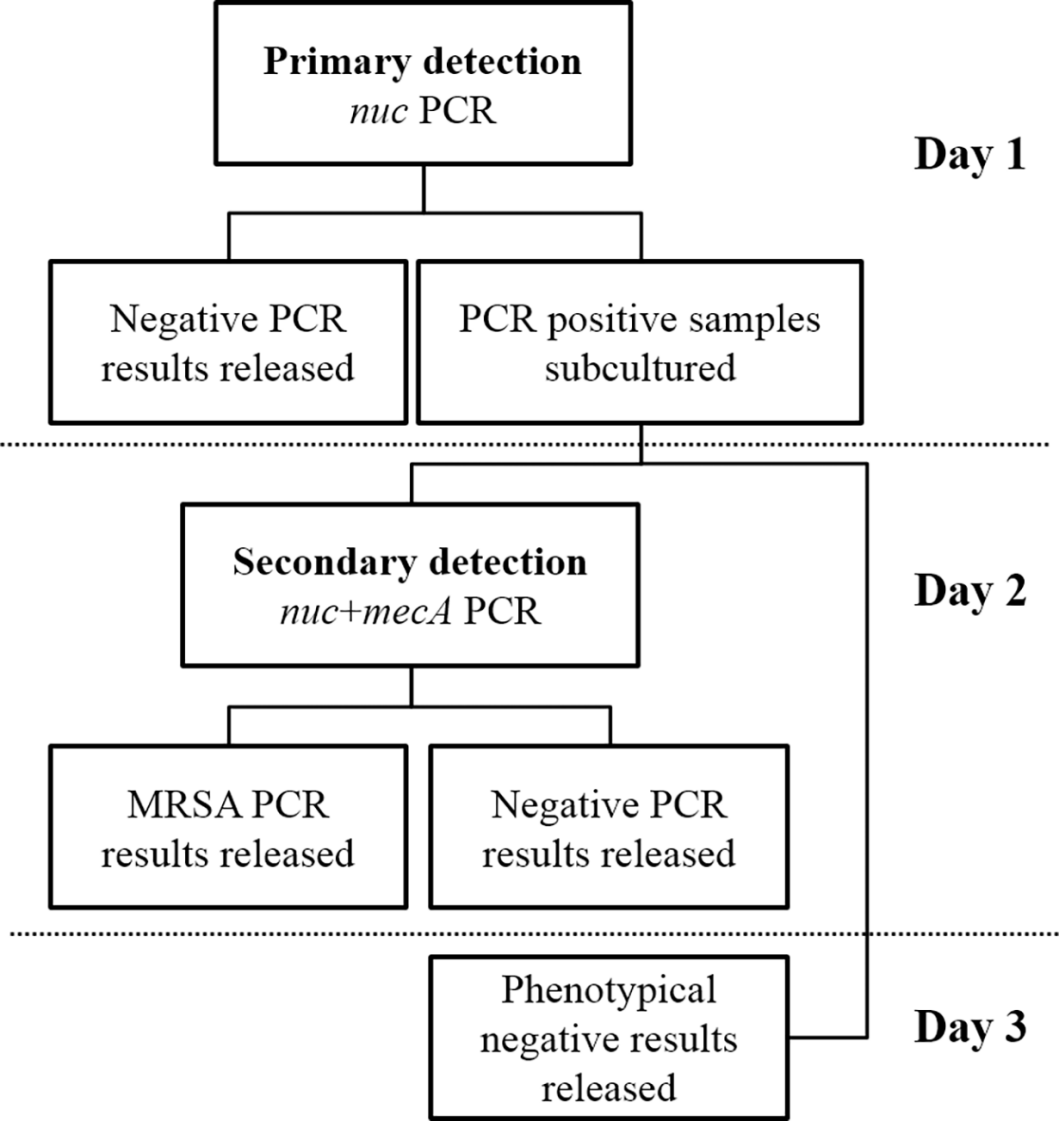
MRSA screening workflow. After selective pre-enrichment in overnight broth, sample cultures are screened for *nuc* presence in the primary detection step (Day 1). Negative results are released the same day and sample cultures positive for *nuc* are subcultivated on selective agar plates. The following day (Day 2), phenotypical MRSA colonies from subcultivated material are by PCR analyzed for presence of both *nuc* and *mecA* genes in a secondary detection step. MRSA positive as well as negative results from this PCR are released the same day. On the following day (Day 3), negative results from non-MRSA phenotypical samples are released.

The approach at Karolinska University Laboratory has been to focus on high sensitivity in a screening process divided in two separate detection steps (Fig 1). In the primary detection step, a PCR targeting the stable standard *S*. *aureus* gene marker *nuc* is performed on DNA from pre-enriched samples as previously described [2, 3, 10]. With this approach, *nuc* negative results (85–90%) are released the same day and sample cultures positive for *nuc* are subcultivated on selective agar plates. In the secondary detection step, MRSA is identified in a PCR targeting both *nuc* and *mecA* genes on phenotypical MRSA colonies from subcultivated material. Molecular detection of the highly conserved *S*. *aureus nuc* gene together with *mecA* from culture is a very sensitive and specific method of detecting MRSA.

The *mecA* gene is not targeted in the primary detection step due to the possibility of the gene being harbored by coagulase-negative staphylococci, a common occurrence in screening samples. The emergence of the alternative resistance gene *mecC* [11] also emphasizes the limitations of strategies based on screening for *mecA* in the primary detection. MRSA harboring *mecC* are detected by phenotypic resistance to cefoxitin and are genotypically confirmed at the Public Health Agency of Sweden by PCR.

The high and steadily increasing sample volumes in the laboratory required a more time-efficient PCR in the primary detection step. By exchanging the detection reagent from SybrGreen (which requires a post amplification melting point step to correctly assess specificity) to hydrolysis probe, the melting point step could be removed. This reduction in PCR reaction time would increase our daily sample capacity with 40%. However, a *nuc* hydrolysis probe PCR would require novel *nuc* oligonucleotides with a reduced amplicon size, from the current 276 base pairs.

Oligonucleotides used in the SybrGreen PCR assay have in general shown high specificity and sensitivity for *S*. *aureus*. However, for one MRSA strain, isolated from a screening sample at our laboratory, the sensitivity for *nuc* was profoundly reduced. Studies revealed that this strain belonged to the clonal complex 75 (CC75), *S*. *aureus* strains that within the genome are 10% divergent compared to the typical *S*. *aureus* [12]. At the time of this study, in 2012–2013, the strain was known solely as *S*. *aureus* CC75, but was later re-classified as *Staphylococcus argenteus* [13].

Herein, we introduce a new *nuc* targeted hydrolysis probe PCR to replace the SybrGreen PCR in the primary detection step of the MRSA screening workflow, described in Fig 1. The aim was to increase the sample capacity while maintaining a low cost. Besides upholding present high specificity and sensitivity for MRSA, the hydrolysis probe PCR also needed to include, but not differentiate, detection of the CC75-lineage of *S*. *aureus/S*. *argenteus* with high sensitivity.

## Materials & methods

### Bacterial strains, clinical samples and culture conditions

Reference strains have been collected from the American Type Culture Collection (ATCC), the Culture Collection, University of Gothenburg (CCUG) and the National Collection of Type Cultures (NCTC).

*S*. *aureus* strains used in this study (n = 25) consisted of a combination of reference strains and clinical isolates. The reference strains (n = 14) were as follows: ATCC25923, ATCC29213, ATCC6538, ATCC700699, CCUG31966, CCUG4151, CCUG41582, CCUG46147, CCUG29318, CCUG32115, CCUG32607, CCUG33290, CCUG33735 and CCUG46923. Clinical strains isolated from wound, nose, throat, urine, hair follicle, abscess, blood and sputum (n = 9), provided by the department of Clinical Microbiology at Örebro University Hospital, were also tested. The remaining two clinical isolates, isolated from wound in 2003 and throat swab in 2007, originated from the department of Clinical Microbiology at Karolinska University Hospital (identified as MRSA using the workflow in Fig 1). One of these isolates was MRSA strain CC75-08, a CC75 lineage/*S*. *argenteus* strain.

A comprehensive study of organisms other than *S*. *aureus* (n = 35) were tested for specificity. For strain information, see S1 Table.

A total number of 629 clinical samples sent to our laboratory for MRSA screening in 2013, originating from swabs from wound, nose, throat, perineum, skin, injection sites, urine, ostomy and catheter sites, were tested for *nuc* presence. Samples were inoculated in a selective broth containing Isosensitest broth 2.3% (ThermoFisher, Basingstoke, UK), Aztreonam 8μg/mL (Sigma-Aldrich, Schnelldorf, Germany), Cefoxitin 4 μg/mL (Sigma-Aldrich) and Colistin 8 μg/mL (Sigma-Aldrich), followed by incubation overnight at 35°C [2, 3, 10] prior to DNA extraction.

Sample cultures positive for the *nuc* gene in the primary detection step were subcultured on SAID CHROMagar^TM^ plates (CHROMagar, Paris, France) and complemented with a cefoxitin disc (30μg, ThermoFisher) in the primary streak. The plates were incubated at 35°C overnight before DNA isolation and molecular detection of *nuc* and *mecA* from single colonies.

For determination of colony-forming units (CFU) in sensitivity tests, bacterial suspensions were plated on 5% blood agar plates and incubated at 35°C overnight before counting.

### DNA extraction

#### Manual extraction

DNA from bacterial strains was isolated using manual heating extraction. Colonies were resuspended in nuclease free water and DNA was then extracted by heating the bacterial suspensions to 94°C for 15 minutes in a block heater. The cell remnants were pelleted by centrifugation at 14000 rpm, leaving the template DNA in the supernatant.

#### Automated extraction, BioRobot M48

The BioRobot M48 using the MagAttract DNA mini M48 kit (both from Qiagen, Hilden, Germany) was used for specificity and sensitivity studies. DNA extraction, based on silica coated magnetic beads, was performed according to the manufacturer’s instructions.

#### Automated extraction, Xiril robot

For clinical sample broth cultures, DNA was isolated using automated extraction. Broths incubated overnight were extracted using the four-channeled Xiril X100 robot or the eight-channel Xiril Neon100 robot (Xiril AG, Hombrechtikon, Switzerland). The same extraction protocol was used for both robots, starting with broth removal by filtration using styrene Multiscreen HTS Filter Plates (Merck Millipore, Billerica, MA, USA). The cells were thereafter resuspended in water and lysed by heating the cell suspensions to 94°C for 15 minutes. DNA template was obtained by a second filtration step separating DNA from cell remnants.

### Real-time PCR

#### Primary detection oligonucleotides

All oligonucleotides used in this study, presented in Table 1, were ordered with a purity of cartridge grade or higher.

**Table 1:** *nuc* targeted oligonucleotides.

Detection format	Primer/ probe	Sequence	Length (nt)	Amplicon size (nt)	Reference
SybrGreen	*nuc*-SG-F	5’- GCGATTGATGGTGATACGGTT-3’	21	276	[2]
	*nuc*-SG-R	5’-CAAGCCTTGACGAACTAAAGC-3’	21		
Hydrolysis probe	*nuc*-F	5’-CCTGAAGCAAGTGCATTTACGA-3’	22	69	This study
	*nuc*-R1	5’-CCTTTGTCAAACTCGACTTCAATTT-3’	25		
	*nuc*-R2	5’-CCTTTGTCAAATTCCACTTCTATTT-3’	25		
	*nuc* probe	5’-FAM-ATGGTAGARAATGC-MGB-3’	14		

#### Primary detection amplification conditions

Amplification of the *nuc* gene using SybrGreen was carried out in a 20 μL reaction mix using 2x Fast SybrGreen Master Mix (ThermoFisher), 0,5 μM of each *nuc*-SG primer (Eurogentec, Seraing, Belgium) and 5 μL of DNA template. A two-step real-time PCR was performed using Fast ABI 7500 (ThermoFisher) with a preincubation step at 95°C for 20 s, followed by 35 cycles (50 cycles in sensitivity testing) of 95°C for 3 s and 60°C for 20 s. The PCR was completed with a melting point analysis step ramping from 60°C to 95°C.

Amplification in hydrolysis probe PCR was carried out in a 20 μL reaction mix using 2x TaqMan Fast Advanced Master Mix (ThermoFisher), 0,5 μM of each *nuc* primer (Eurogentec), 0,2 μM of *nuc* probe (ThermoFisher) and 5 μL of DNA template. A two-step real-time PCR was performed using Fast ABI 7500 (ThermoFisher) starting with an UNG-step at 50°C for 2 min and a pre-incubation step at 95°C for 20 s, followed by 40 cycles (50 cycles in sensitivity testing) of 95°C for 3 s and 60°C for 30 s.

#### Secondary detection oligonucleotides and amplification conditions

*nuc*-SG primers used in the secondary detection were the same as in the primary detection using SybrGreen (Table 1). Primer sequences used for *mecA* amplification have been previously described [14].

Amplification of *nuc* and *mecA* was performed in parallel in the PCR instrument, but in separate reactions. The reaction mix of 20 μL contained 2x RotorGene SybrGreen (Qiagen), 0,5 μM of primers (Eurogentec), directed at *nuc* and *mecA*, and 5 μL of DNA template. A real-time PCR was performed in RotorGene 3000 (Qiagen) starting with a pre-incubation step at 95°C for 5 min, followed by 35 cycles of 95°C for 5 s and 60°C for 15 s. The amplification was followed by a melting curve analysis with a ramp from 60°C to 95°C.

### 16S detection

Presence of extracted DNA from bacterial strains other than *S*. *aureus* (S1 Table) was analyzed by amplification of the 16S rDNA gene. Primers and amplification conditions have been previously described [15]. DNA presence was confirmed by analyzing quantification cycle (Cq) values and melting peaks of the amplified 16S gene.

### DNA sequence analysis

#### Sanger sequencing

Pre-sequencing amplification was performed from CC75-08 strain DNA using *nuc* oligonucleotides according to the secondary detection SybrGreen PCR protocol (described in previous section). Post-amplification steps, including protocol for DNA purification and Sanger sequencing of the *nuc* gene, is previously described [15]. The *nuc* sequences obtained were first analyzed in SeqScape Software 3 version 3.0 (ThermoFisher) for species confirmation and subsequently analyzed by Nucleotide BLAST (Basic Logic Alignment Search Tool, NCBI, https://blast.ncbi.nlm.nih.gov/) and compared to open Gene Bank sequences.

#### Whole genome sequencing

The genome of selected clinical isolates from the method comparison study was analyzed with whole genome sequencing. Bacteria were pretreated by lysozyme (9.5 mg/mL) and lysostaphin (0.24 mg/mL) followed by extraction of genomic DNA with the EZ1 Advanced XL system using the EZ1 DNA Tissue Kit (both from Qiagen). Extracted DNA was sent to a sequencing core facility (SciLife Lab, Solna, Sweden) and sequenced using a standard operating procedure for Illumina HiSeq 2500 (Illumina, San Diego, CA, USA).

In selected cases the *nuc* gene was mapped in silico using CLC Genomics Workbench 11 (Qiagen Bioinformatics, Aarhus, Denmark). Alignments were conducted with *nuc* genes from different strain sequences and the forward and reverse primers using software BioEdit Sequence Alignment Editor software, version 7.2.1 (Ibis Therapeutics, Carlsbad, CA, USA).

In silico analysis of whole genome sequences for multi locus sequence typing (MLST) and toxin genes was performed on selected clinical isolates using software from Center for Genomic Epidemiology [16].

### Experimental setup

#### Oligonucleotide design

For primer and probe design, the following software were used; Primer Express, version 3.0.1 (ThermoFisher) and Oligo Analyzer, version 3.1 (https://eu.idtdna.com/calc/analyzer). For the hydrolysis probe PCR oligonucleotide design, 40 unique *nuc* sequences from *S*. *aureus* strains were obtained from the NCBI database (https://www.ncbi.nlm.nih.gov/nucleotide) and aligned in the BioEdit software (S1 Fig). The primer and probe sequences were complementary within the conserved regions of the *nuc* gene. In addition, 24 unique *nuc* sequences from *S*. *argenteus* strains (whereof two are defined as *S*. *aureus* in the NCBI database, but show higher resemblance to *S*. *argenteus* sequences) are presented in S2 Fig.

In experiments for oligonucleotide composition, CC75-08 tests in the hydrolysis probe PCR were performed in duplicate, while CCUG31966 data was generated from single testing.

#### Sensitivity study

The hydrolysis probe PCR was tested for sensitivity in comparison with the SybrGreen PCR assay. PCR was performed on DNA from CCUG31966 and the CC75-08 strain in 10-fold dilutions.

In the sensitivity test when comparing the two PCR assays in *S*. *aureus* DNA serial dilutions, both SybrGreen PCR and hydrolysis probe PCR were performed in 50 rounds of amplification and in duplicate. PCR efficiency (E) was calculated using the formula $E=10-\frac{1}{slope}-1$.

#### Specificity study

Presence of extracted DNA from bacterial strains in the specificity study was confirmed by 16S amplification while DNA from viral strains in specificity study was confirmed positive by directed real-time PCR. Presence of DNA from yeast strain was not confirmed.

#### Method comparison study

While assessing the assay performance in clinical samples, the two primary *nuc* PCR assays (SybrGreen and hydrolysis probe) were tested in parallel in a method comparison study. For the purpose of facilitating ΔCq calculations between the two primary detection PCR assays, the hydrolysis probe PCR was run at 40 cycles, while the SybrGreen PCR was run at 35 cycles (as previously optimized).

## Results

### Separate reverse primers and a degenerated probe are required for specific amplification

**Fig 2:**
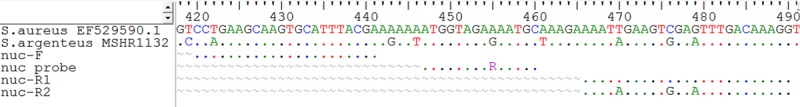
Hydrolysis probe PCR oligonucleotides complementary to *nuc* gene. Alignments were performed on the *nuc* gene in *S*. *aureus* EF529590.1 and in CC75 lineage/*S*. *argenteus* MSHR1132. Separate reverse primers were designed to enable complement to both aligned *nuc* sequences.

During the initial part of the study, a strain from a clinical sample showed difficulties in *nuc* amplification, using a variety of primer combinations. A region of the *nuc* gene was sequenced by Sanger sequencing and analyzed using the NCBI nucleotide database. The sequenced *nuc* region from the clinical strain (in this manuscript named CC75-08) showed 99% similarity with the *nuc* gene in MSHR1132, a *S*. *aureus* CC75 lineage strain, later named *S*. *argenteus*. By aligning the *nuc* sequences of *S*. *aureus* and MSHR1132, oligonucleotides could be designed to fit all aligned sequences (Fig 2, EF529590.1 representing *S*. *aureus* and MSHR1132 representing the CC75 lineage*/S*. *argenteus*). All aligned sequences are illustrated in S1 and S2 Figs.

The designed *nuc*-F primer mismatched in position 3 at the 5’-end against the *nuc* gene in MSHR1132, but was identical in sequence with other aligned *nuc* sequences. The probe was designed degenerated in one position. As the *nuc* sequence at the location of the reverse primer revealed high sequence variation, separate reverse primers were designed; specific for *S*. *aureus* (*nuc*-R1) and specific for the CC75 strain (*nuc*-R2). The sequence of the two reverse primers differs at three nucleotide positions.

**Fig 3:**
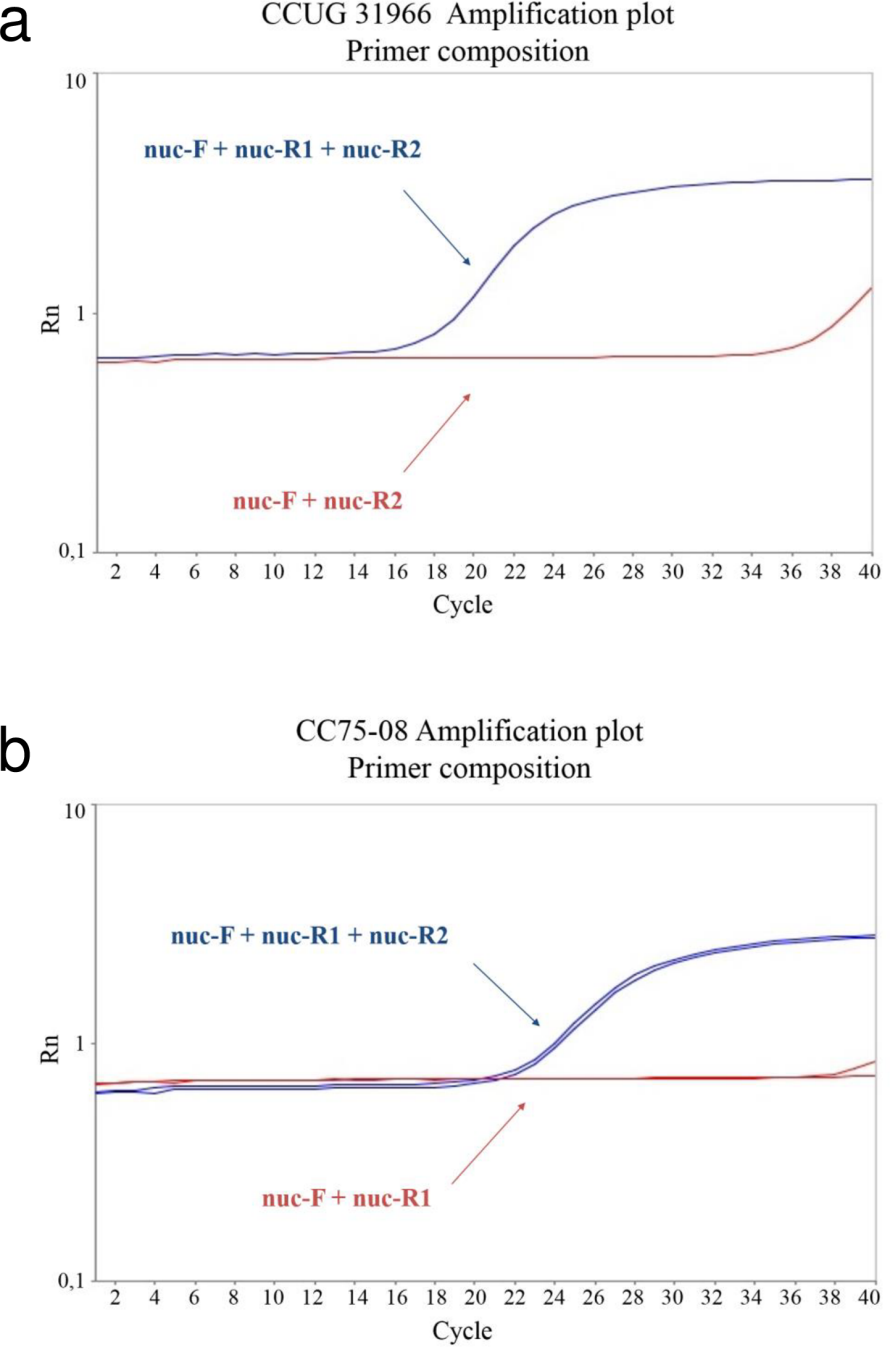
*Nuc* amplification of CCUG31966 and CC75-08 achieved in hydrolysis probe PCR. (a) *S*. *aureus* (CCUG31966) amplified with *nuc*-F+*nuc*-R1+*nuc*-R2 and *nuc*-F+*nuc*-R2. (b) CC75 lineage strain/*S*. *argenteus* (CC75-08) amplified with *nuc*-F+*nuc*-R1+*nuc*-R2 and *nuc*-F+*nuc*-R1.

**Fig 4:**
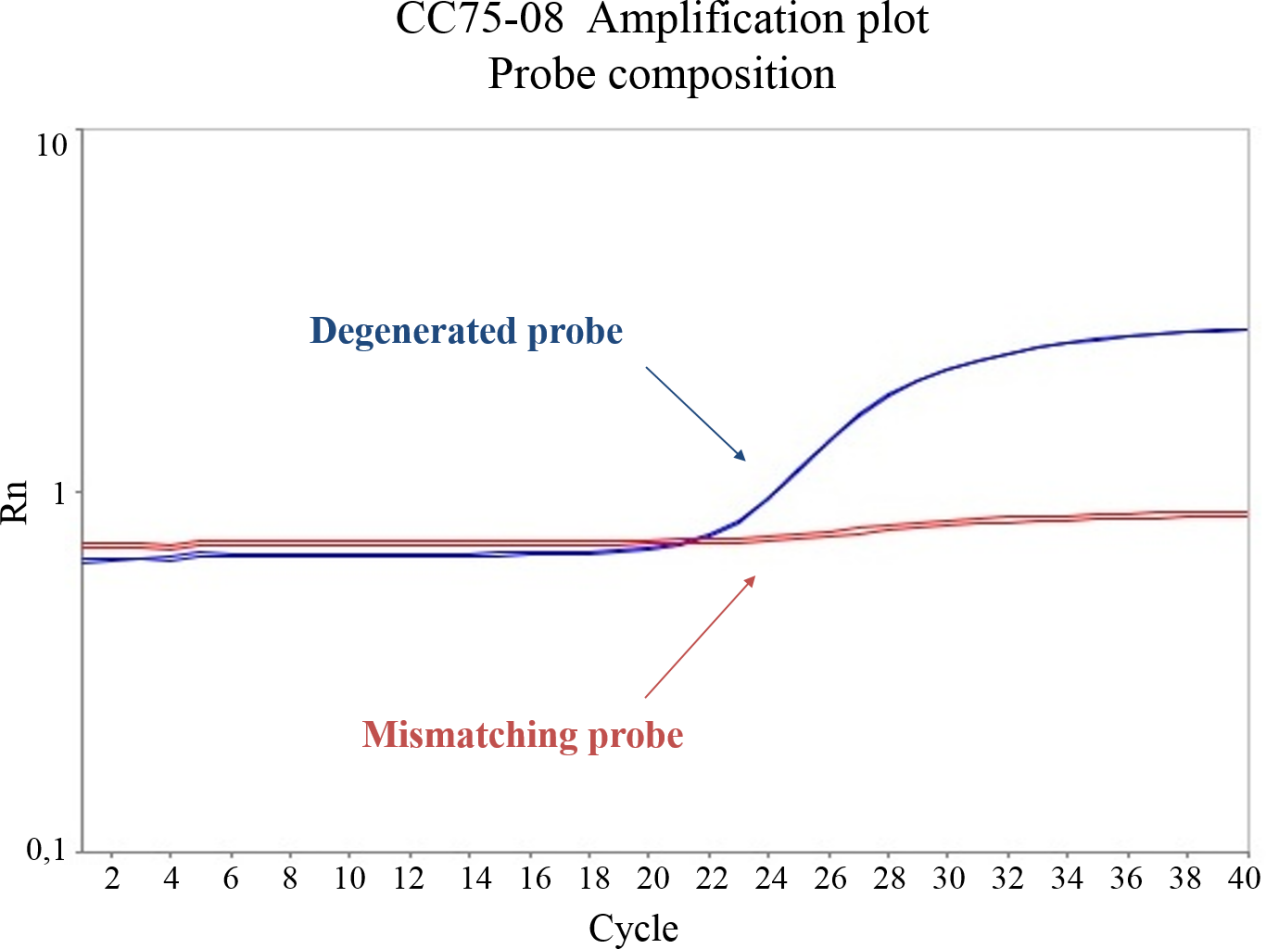
*Nuc* amplification of CC75-08 detected by degenerate probe in hydrolysis probe PCR. *Nuc* amplification detection of CC75 lineage strain/*S*. *argenteus* (CC75-08) by degenerate probe and probe mismatching in one position. Amplification was performed using primers *nuc*-F, *nuc*-R1 and *nuc*-R2.

In the hydrolysis probe PCR, reverse primer *nuc*-R1 was shown to be specific and necessary for amplification of *S*. *aureus* strain CCUG31966, while *nuc*-R2 had little effect on *nuc* amplification (Fig 3A). The opposite was observed for the CC75-08 strain (Fig 3B). DNA amplification in both CCUG31966 and CC75-08 was successfully detected by the degenerate *nuc* probe. The *nuc* sequence in CCUG31966 and CC75-08 differ only at one position in the designed probe region. A degenerate probe was designed to obtain an adequate fluorescence from both strains during amplification (Fig 4).

As a result of these findings, all further experiments utilizing the hydrolysis probe PCR were performed with the following oligonucleotide composition: *nuc*-F, *nuc*-R1, *nuc*-R2 and *nuc* probe (as specified in Table 1). This setup would include, but not differentiate, CC75-lineage strains/*S*. *argenteus* in the primary detection step.

### Increased sensitivity for CC75 lineage strain/*S*. *argenteus* with the hydrolysis probe PCR

All *S*. *aureus* strains (n = 25), extracted manually, were detected by the oligonucleotides in the primary hydrolysis probe PCR. None of the non-*S*. *aureus* bacterial strains (n = 29), viral strains (n = 5) or the yeast strain (n = 1), all extracted on the BioRobot M48 with DNA presence confirmed by 16S rDNA amplification or directed real-time PCR, were detected by hydrolysis probe PCR.

**Fig 5:**
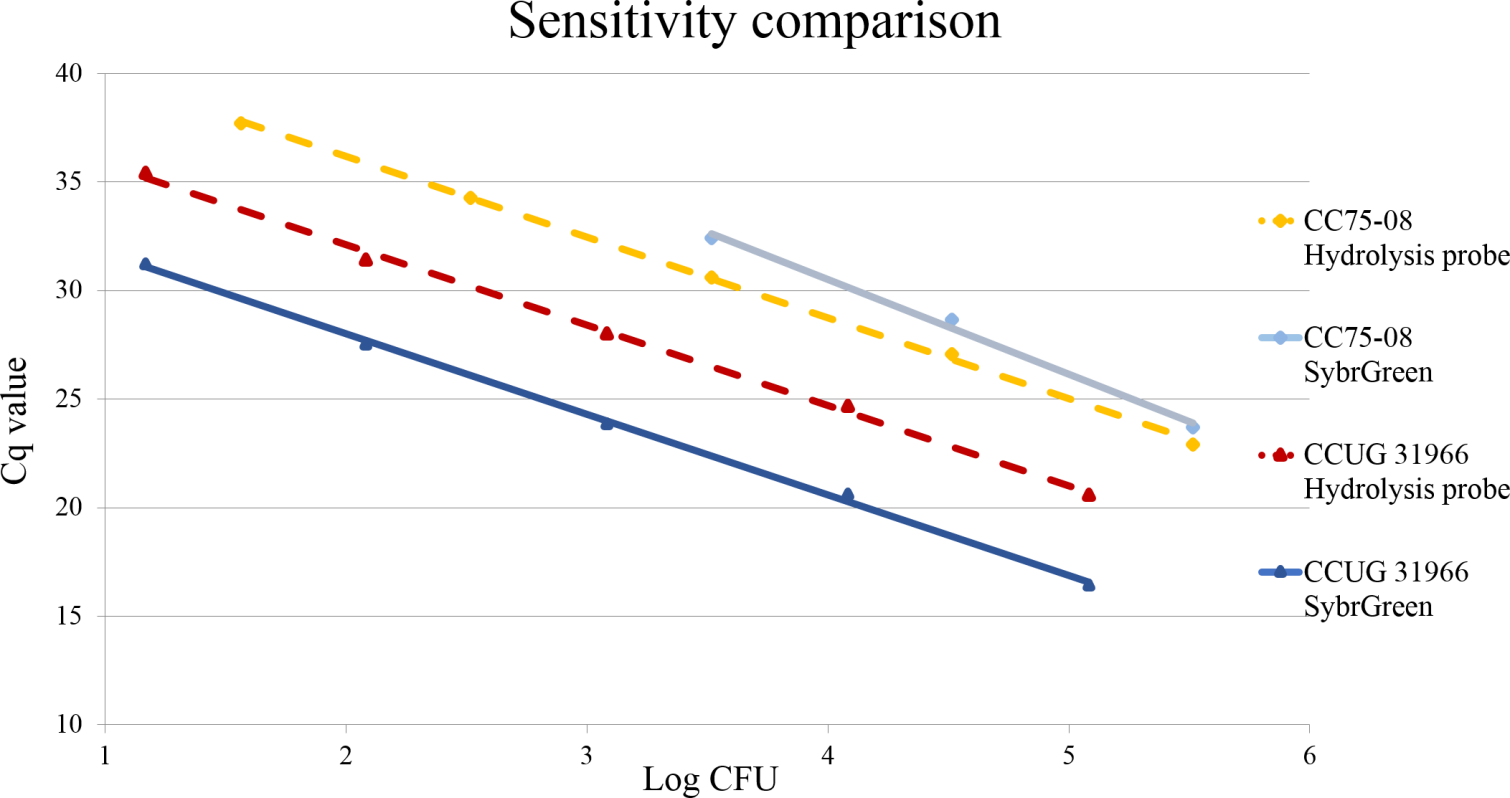
Increased PCR sensitivity for CC75-08 in the hydrolysis probe PCR. Analytical sensitivity equal for *S*. *aureus* (CCUG31966) in hydrolysis probe PCR and SybrGreen PCR. Analytical sensitivity increased for CC75 lineage/*S*. *argenteus* (CC75-08) strains in hydrolysis probe PCR.

Plotting the Cq values against the log_10_ CFU values illustrated that the PCR reaction for the primary hydrolysis probe PCR was linear over at least five orders of magnitudes for CCUG31966 and CC75-08 (Fig 5). The lowest CFU detected per PCR reaction and the PCR efficiency were similar for CCUG31966 in the two PCR assays. However, for CC75-08, both lowest CFU detected/PCR reaction and PCR efficiency increased considerably when amplified with the hydrolysis probe PCR (Table 2).

**Table 2:** Calculated PCR efficiency and lowest CFU detected/PCR reaction.

		Primary SybrGreen PCR	Hydrolysis probe PCR
**PCR efficiency, %**	CCUG31966	86	86
	CC75-08	70	86
**Lowest CFU/PCR reaction**	CCUG31966	1,5E+01	1,5E+01
	CC75-08	3,3E+03	3,7E+01

### Increased detection of *nuc* and MRSA in clinical samples with hydrolysis probe PCR

By exchanging detection format from SybrGreen to hydrolysis probe, the melting curve step in the PCR could be omitted. Each PCR-reaction could thereby be shortened 20 minutes.

In the method comparison study, primary detection *nuc* PCR was performed with the SybrGreen PCR and the hydrolysis probe PCR in parallel on 629 clinical samples. The hydrolysis probe PCR, run at 40 cycles, was analyzed twice, with a Cq cut-off at 40 and at 37 PCR cycles, and then compared to the SybrGreen PCR with a Cq cut-off at 35 (Table 3). The *nuc* positive share for clinical samples was 10% for the SybrGreen PCR, 11% for the hydrolysis probe PCR with a Cq cut-off at 37, and 15% for the hydrolysis probe PCR with a Cq cut-off at 40.

**Table 3:** Results for primary PCR detection and culture for clinical samples.

	SybrGreen PCR	Hydrolysis probe PCR	
Cq cut-off	35	37	40
*nuc* positive	64 (10%)	70 (11%)	91 (15%)
MRSA in culture	4 (0,6%)	5 (0,8%)	6 (1,0%)

All clinical samples positive for *nuc* in at least one assay were subcultured. Culture showed MRSA presence in four clinical samples that were *nuc* positive in both SybrGreen and hydrolysis probe PCR Also, culture revealed MRSA in two additional clinical samples, originating from the same patient, with Cq values 33 and 39 in the hydrolysis probe PCR and undetected by the SybrGreen PCR. Further studies revealed that the *nuc* genes in these MRSA isolates were amplified using the R1 reverse primer.

### Analysis of whole genome sequencing data

Whole genome sequencing was later performed on in total ten clinical MRSA isolates. One of these isolates was included in the comparison study and only detected only by the hydrolysis probe PCR, not by the SybrGreen PCR. MLST revealed that the sequence type (ST) of this isolate was 30 and it carried the virulence gene toxic shock syndrome toxin 1 (*tss-1*). The *nuc* sequence of this isolate was further analyzed and aligned together with the hydrolysis probe PCR oligonucleotides. The alignment revealed a mismatch (G_239_ → T) in the *nuc*-SG-F primer binding region, four nucleotides from the 3’-end. The *nuc* sequence was complementary to reverse primer *nuc*-R1 and no mismatches were found.

MLST was also performed on strain CC75-08 as well as eight isolates diagnosed as MRSA in 2013–2016 in which the *nuc* genes were amplified with the *nuc*-R2 primer (specific for the CC75 lineage/*S*. *argenteus*). Three different sequence types were observed: ST1223 (n = 5, includes CC75-08), ST2250 (n = 2) and ST2793 (n = 2), all of which belong to the CC75 lineage/*S*. *argenteus* as expected. The *nuc* sequence of one selected strain (ST1223) was further analyzed and aligned together with the hydrolysis probe PCR oligonucleotides. The *nuc* sequence was complementary to reverse primer *nuc*-R2 and no mismatches were found.

### Increased stability with hydrolysis probe PCR

**Fig 6:**
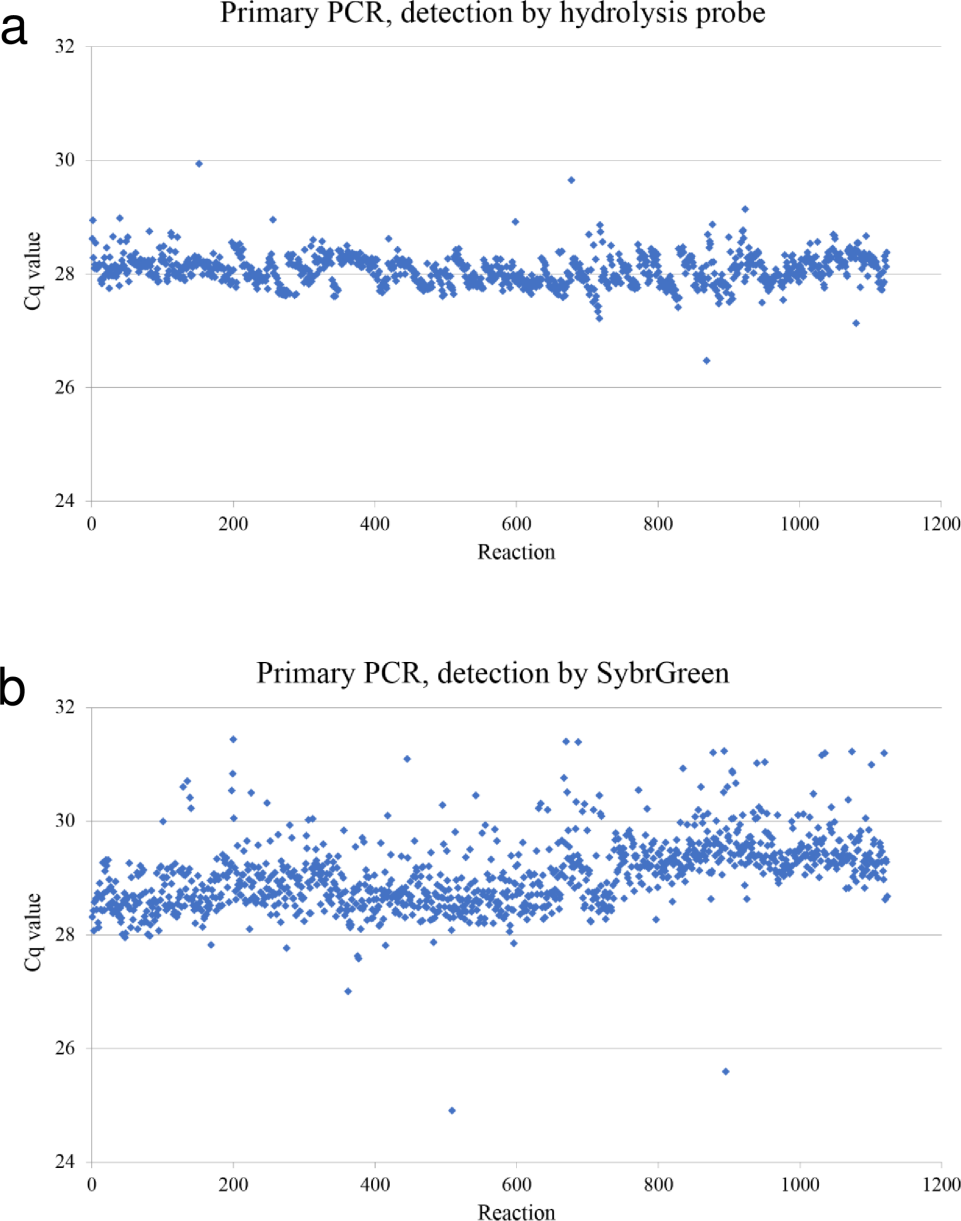
Increased stability using hydrolysis probe PCR. Cq values from PCR control collected during one year from hydrolysis probe PCR (a) and SybrGreen PCR (b) show increased stability in hydrolysis probe PCR.

For both primary detection *nuc* PCR assays, PCR control Cq values (originating from CCUG31966) from over 1100 PCR reactions were documented. The plotted Cq values show an increased analytical stability for the hydrolysis probe PCR (Fig 6A) compared to primary SybrGreen PCR (Fig 6B). The coefficient of variation was low in both assay formats, 1.69 for the hydrolysis probe PCR versus 2.18 in the SybrGreen assay.

## Discussion

Herein, we describe the introduction of a new primary detection step *nuc* PCR, including novel oligonucleotides and hydrolysis probe. The assay allows screening of high sample volumes at a low cost with the ability to include or exclude detection of CC75 *S*. *aureus*/*S*. *argenteus*.

The final results from the screening positive samples are obtained after two further incubation days, the screening positive non-MRSA samples should therefore be kept to a minimum. In the method comparison study, the quantity of screening positive samples in the hydrolysis probe PCR varies considerably with the Cq cut-off. By lowering the Cq cut-off from 40 to 37, we could decrease the quantity of *nuc* positive screening samples by 25% and still maintain a high MRSA sensitivity.

In the method comparison study, the hydrolysis probe PCR detected two MRSA positive clinical samples, from the same patient, that were negative in the SybrGreen PCR. The fact that the *nuc* genes in these MRSA isolates were amplified using the R1 reverse primer indicates that these strains are distinct from the CC75 lineage/*S*. *argenteus*. A mismatch found in the *nuc*-SG-F primer binding region is the probable cause for the reduced SybrGreen PCR sensitivity towards this strain.

Despite the benefits described for the hydrolysis probe PCR, some limitations remain. The high sensitivity comes at the expense of an increased number of positive PCR results in primary detection step compared to the SybrGreen PCR. Furthermore, even though overall sensitivity was increased, the PCR still fails to detect the rare [17] cases of *nuc* negative MRSA strains.

The CC75 complex describes a lineage with 90% shared genome with *S*. *aureus*. It has been suggested that this lineage should be considered as a novel species, *S*. *argenteus* [13] and clinical differences have been established [18]. *S*. *argenteus* has spread internationally and share many of *S*. aureus virulence genes [19]. The CC75-08 strain is defined as *S*. *aureus* by standard *nuc* primers [13], [this study], yet with reduced sensitivity. We have in our laboratory also found that the commercial SCC*mec* based test, Xpert MRSA NxG (Cepheid, Sunnyvale, CA, USA), identifies the CC75-08 strain as an MRSA. Only a few new patients are each year diagnosed with MRSA with this this lineage/species at our laboratory. The various sequence types ST1223, ST2250 and ST2793 that were defined for these strains have all previously been reported in known *S*. *argenteus* strains [18, 19] which shows that several different strains of the CC75 lineage/*S*. *argenteus* are in circulation in Sweden.

Currently, discussions are held in Sweden regarding the inclusion or exclusion of *mecA* positive bacteria from the CC75 lineage/*S*. *argenteus* in the MRSA definition used in the Communicable Disease Act. There is no current international consensus in this matter. In either case, most current MRSA diagnostic assays in routine practice should either increase CC75 sensitivity or omit it from the assay completely. In the assay described in this manuscript, both alternatives are possible by simply adjusting the presence of reverse primers and/or probe sequence. If desired, the assay can also easily distinguish *S*. *argenteus* from *S*. *aureus* in PCR detection by replacing the degenerated probe with specific probes with separated emission wavelengths.

By the introduction of a hydrolysis probe PCR, we are able to increase our daily MRSA screening sample capacity by 40%, up to 637 samples per day and analysis platform, with a maintained assay specificity and sensitivity. This is done at a low cost and with an assay that provides higher PCR stability, an important factor for a diagnostic laboratory in order to provide a high quality in diagnostics and a minimum of re-testing. This assay is thereby especially suitable for clinical laboratories with high sample volumes. Also, the current oligonucleotide composition increases the analytical sensitivity for the CC75 lineage/*S*. *argenteus* considerably. By minor oligonucleotide rearrangements, the assay can be customized to distinguish or completely omit CC75 lineage/*S*. *argenteus* detection, thus ensuring the accuracy of the MRSA diagnostic assay.

## Supporting information

S1 Fig Alignment for hydrolysis probe PCR oligonucleotides with *nuc* gene with 40 *S*. *aureus* strains. *Nuc* sequences are complementary to hydrolysis probe PCR oligonucleotides comprising the *nuc*-R1 reverse primer.
(TIF)

S2 Fig Alignment for hydrolysis probe PCR oligonucleotides with *nuc* gene with 24 *S*. *argenteus* strains. *Nuc* sequences are complementary to hydrolysis probe PCR oligonucleotides comprising the *nuc*-R2 reverse primer.
(TIF)

S1 Table Reference strains used to test specificity. (DOCX)
