# Supplementary figures and images for: Introduction of a hydrolysis probe PCR assay for high-throughput screening of methicillin-resistant Staphylococcus aureus with the ability to include or exclude detection of Staphylococcus argenteus
Source: PLoS One. 2018 Feb 9;13(2):e0192782. doi: 10.1371/journal.pone.0192782 (PMC5806904; doi:10.1371/journal.pone.0192782)

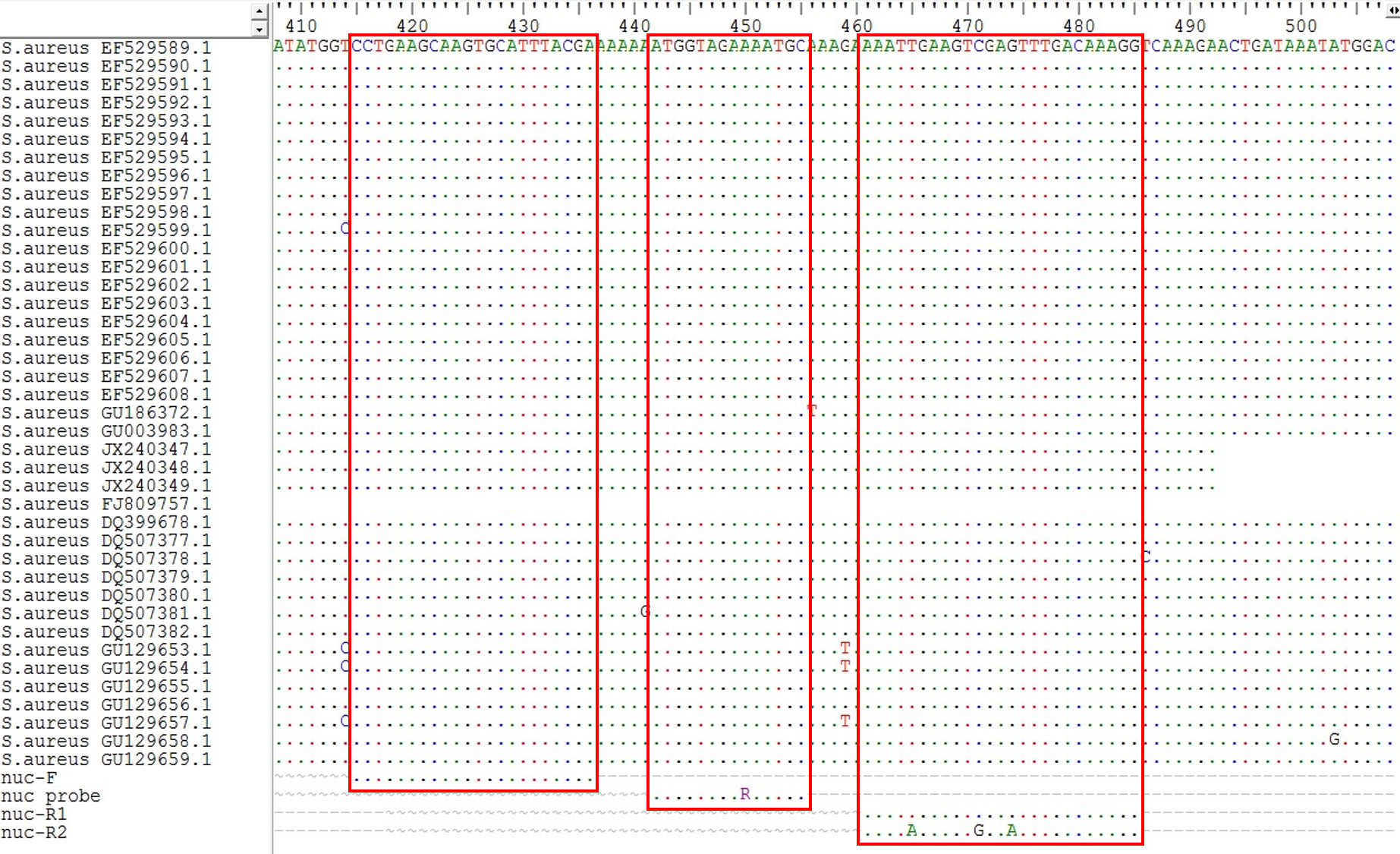

Supplement: S1 Fig — Nuc sequences are complementary to hydrolysis probe PCR oligonucleotides comprising the nuc-R1 reverse primer. (TIF) [file pone.0192782.s001.tif]

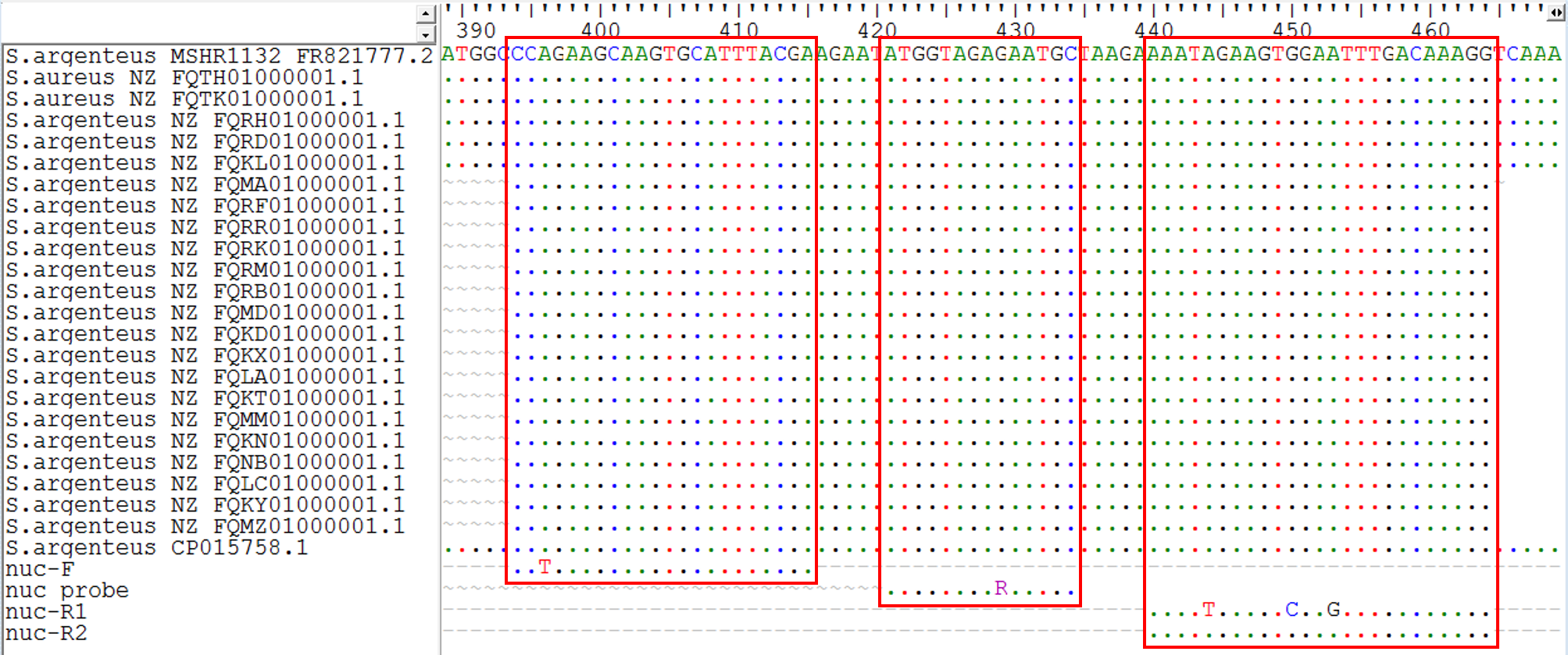

Supplement: S2 Fig — Nuc sequences are complementary to hydrolysis probe PCR oligonucleotides comprising the nuc-R2 reverse primer. (TIF) [file pone.0192782.s002.tif]
