# Supplementary material for: Introduction of a hydrolysis probe PCR assay for high-throughput screening of methicillin-resistant Staphylococcus aureus with the ability to include or exclude detection of Staphylococcus argenteus
Source: PLoS One. 2018 Feb 9;13(2):e0192782. doi: 10.1371/journal.pone.0192782 (PMC5806904; doi:10.1371/journal.pone.0192782)
Supplement: S1 Table — (DOCX) [file pone.0192782.s003.docx]

# Supporting information

**S1 Table.**

| **Bacteria strains** | **Strain ID** |
| --- | --- |
| *Streptococcus pyogenes* | ATCC19615 |
| *Streptococcus canis* | CCUG27661 |
| *Streptococcus agalactiae gr B* | CCUG4208 |
| *Streptococcus equisimilis* | CCUG4211 |
| *Enterococcus faecalis* | ATCC29212 |
| *Staphylococcus epidermidis* | CCUG18000A |
| *Streptococcus milleri* | NCTC10708 |
| *Streptococcus pneumoniae* | CCUG33638 |
| *Haemophilus influenzae* | CCUG23946 |
| *Haemophilus parainfluenzae* | CCUG12836 |
| *Enterococcus faecium* | CCUG542 |
| *Staphylococcus lugdunensis* | CCUG25348 |
| *Pseudomonas aeruginosa* | ATCC27853 |
| *Bacillus cereus* | CCUG7414 |
| *Bacillus subtilis* | CCUG163 |
| *Legionella pneumophila sg.1* | ATCC33152 |
| *Legionella micdadei* | ATCC33218 |
| *Edwardsiella tarda* | CCUG33985 |
| *Providencia rettgeri* | CCUG33987 |
| *Proteus mirabilis* | ATCC29906 |
| *Enterobacter cloacae* | CCUG33986 |
| *Serratia marcescens* | CCUG33988 |
| *Staphylococcus saprophyticus* | CCUG3706 |
| *Staphylococcus xylosus* | ATCC29971 |
| *Klebsiella pneumoniae* | ATCC25955 |
| *Escherichia coli* | ATCC25922 |
| *Bordetella pertussis* | ATCC8467 |
| *Bordetella parapertussis* | ATCC15311 |
| *Moraxella catarrhalis* | CCUG18284 |
|  |  |
| **Viral strains** |  |
| Herpes Simplex Virus 1 |  |
| Herpes Simplex Virus 2 |  |
| Varicella Zoster Virus |  |
| Cytomegalovirus |  |
| Human Herpes Virus 6 GS |  |
|  |  |
| **Yeast strain** |  |
| *Candida albicans* | ATCC90028ST |
